# Society Meetings

**Published:** 1899-01

**Authors:** 


### SOCIETY MEETINGS.

THE Medical Society of the County of Chautauqua held its regular semi-annual meeting at Fredonia, December 13, 1898, under the presidency of Dr. M. N. Bemus, of Jamestown, when the following program was observed :

Business session 11.30 o'clock a. m.

Scientific session, 2 o'clock p. m.—(1) Duodenitis, by Dr. V. M. Griswold, vice-president, Fredonia; discussed by Dr. N. E. Beardsley, Dunkirk; MacDonald Moore, Fredonia. (2) Appendicitis, by Dr. J. J. Mahoney, Jamestown. In the absence of Dr. Mahoney this paper was read by Dr. H. A. Eastman; discussed by Dr. T. E. Soules, Westfield; Dr. George F. Smith, Sinclairville. (3) Some reminiscences of early practice, by Dr. L. H. Snow, Jamestown. (4) Compound fractures, by Dr. A. Wilson Dods, Fredonia; discussed by R. T. Rolph, Dunkirk; Dr. E. M. Scofield, Jamestown. (5) Presentation or report of interesting cases.

Evening session—(5) Importance of middle ear inflammation and its treatment, by Dr. G. E. Blackham, Dunkirk. Illustrated with lantern slides.

The following-named physicians were admitted to membership : C. H. Richards, of Dunkirk; A. Austin Becker and Fred C. Hunt, of Jamestown.

The following was unanimously adopted :

*Resolved*, That contract medical service is beneath the recognition of members of this society.

Those present were Drs. M. N. Bemus, V. M. Griswold, N. G. Richmond, MacDonald Moore, A. W. Dods, J. W. Morris, W. D. Wellman, L. H. Snow, W. Stuart, H. A. Eastman, A. Borden, G. E. Blackham, C. H. Richards, N. E. Beardsley, J. J. Drake, C. A. Ellis, and T. D. Strong. The next annual meeting is to be held at Chautauqua, N. Y., the second Tuesday in July, 1899.

---

THE Medical Society of the State of New York will hold its ninety-third annual meeting at Albany, Tuesday, Wednesday and Thursday,

January 31 and February 1 and 2, 1899, under the presidency of Dr. John O. Roe, of Rochester. The business committee, Dr. W. G. Macdonald, chairman, Dr. E. B. Angell and Dr. James M. Winfield, is preparing an elaborate program, which will include a military discussion that will commence promptly at 4 o'clock Tuesday afternoon and continue until the close of that session. Among the subdivisions of topics to be considered under this head are the following: hygienic camps, typhoid fever, modern bullet wounds, cardiac deficiency, sinks, water supply and sewage. It is expected that a number of physicians, engineers and others who have been prominent in the military service of the state and nation, as well as sanitarians, officers and physicians, will participate.

It is contemplated that a portion of Tuesday evening will be set apart for the discussion of questions pertaining to the state care of consumptives, lead by Dr. John H. Pryor, of Buffalo. A large number of papers on miscellaneous subjects has been offered to the committee and a meeting of unusual interest appears in prospect.

---

The Medical Society of the County of Erie will hold its seventy-eighth annual meeting at the rooms of the Buffalo Academy of Medicine, Tuesday, January 10, 1899, at 10 o'clock a. m., under the presidency of Dr. Lucien Howe. The business committee is preparing a program that was not issued in season for publication in the Journal, but we are informed that subjects of interest will be presented for consideration.

---

The Roswell Park Medical Club, of Buffalo, at its recent annual meeting elected the following officers for the current year: President, Dr. W. E. Dignen; vice-president, secretary and treasurer, Dr. Hugh McIntyre; critic, Dr. J. C. Thompson. The club holds regular monthly meetings at its rooms in the Mooney-Brisbane building.

---

The Sanatory Club of Buffalo, held its regular bi-monthly meeting in the lecture room of the Buffalo Law School, Ellicott Square, Wednesday evening, December 14, 1898. The topic of the evening was Hygienic camps, presented by the following experts in their respective lines:

The hygienic camp, Henry R. Hopkins, M. D., professor of hygiene, university of Buffalo, president of the club. The camp of instruction, Lieutenant Peter C. Harris, Quartermaster 13th United

States Infantry; discussion opened by Major Thomas W. Symons, Corps of Engineers, U. S. A. Sewers, C. E. P. Babcock, C. E., Assistant City Engineer. Late major 65th N. Y. Volunteers; discussion opened by G. H. Norton, C. E., Assistant City Engineer. Late captain 65th N. Y. Volunteers. Water supply, L. H. Knapp, C. E., Assistant Superintendent and Engineer Bureau of Water. Discussion opened by W. D. Greene, M.D., Deputy Health Commissioner. Garbage and sewage disposal, Edward Clark, M. D. Late member of the committee on disposal of sewage and garbage, American Public Health Association; discussion opened by Eugene Smith, M. D. Late major 65th N. Y. Volunteers. Statistics, Lieutenant Paul B. Malone, 13th United States Infantry; discussion opened by William Warren Potter, M. D., editor of the BUFFALO MEDICAL JOURNAL. Walks and streets, H. C. Gardner, 74 Regiment, N. G. S. N. Y. Firm of Cockburn Brothers & Co., concrete contractors. The relative importance of flies and water supply in spreading disease, M. A. Veeder, M. D., Lyons, New York; discussion opened by A. H. Briggs, M. D. Late major and surgeon 65th N. Y. Volunteers.

With some slight changes, due to inability of referees to attend, the program was carried out. The JOURNAL will publish the full proceedings of this interesting meeting in future editions.

---

THE Southern Surgical and Gynecological Association held its eleventh annual meeting at Memphis, Tenn., and it is regarded as one of the best meetings in the history of the society. A large number of distinguished surgeons and gynecologists were in attendance, and a splendid list of papers were read and discussed.

We quote the Alabama *Medical and Surgical Age* as follows:

The members who attended the meeting are saying many nice things of the profession and people of Memphis for the splendid hospitality accorded them during the meeting of the society.

In connection with the Surgical and Gynecological meeting, an important meeting of the Southern Medical College Association, at which Dr. George A. Ketchum, of Mobile, presided was held and by a unanimous vote it was decided to adopt the four years' course, to take effect January 1, 1899. So all students who enter college after this date will be required to attend four years before being eligible for graduation.

Dr. Richard Douglas, of Nashville, President of the Southern Surgical and Gynecological Association, prefaced his annual address by saying:

"As president of this distinguished body, I am fully conscious of my exalted position and deeply sensible of the friendly spirit and kindly good will that has induced you to so honor me. This association in the ten short years of its history has become renowned for the excellence of its scientific work, the truthfulness of its records and the spirit of warm friendship that pervades its membership. And we cannot too cordially express our thanks to Dr. W. E. B. Davis, our permanent secretary, to whose indefatigable efforts the Southern Surgical and Gynecological Association owes its existence and high standing. Nor are we unmindful of our obligation to our distinguished predecessors, who by their justice, courtesy and dignity in office and wisdom in council have guided our deliberations and smoothed our difficulties.

"Perhaps a true interpretation of my duties of the hour would demand that I address you by recounting our recent deeds of prowess, arranging in chronological order the victories of the allied armies—the science and art of surgery—have won in the great battle against disease and death; or of telling of recent discoveries, new devices and modifications of technique, the territorial acquisitions of our profession. Yet, inasmuch as the greatest latitude is granted me, I prefer to restrict my remarks to a very commonplace subject, but one in which both branches of the association feel a common interest—that of acute general peritonitis.

"A proper appreciation of the time of the association will not permit me to discuss this subject in all its phases; for that reason I shall confine myself to an effort to present to you a workable classification. As an introduction to the subject, I shall first consider the attempts that have been made to form a classification on a bacteriological basis."

Officers elected for the ensuing year were: Dr. Joseph Taber Johnson, of Washington, president; Dr. F. M. Parham, of New Orleans, and Dr. W. L. Robinson, of Danville, Va., vice-presidents, Dr. A. M. Cartledge, of Louisville, treasurer, and W. E. B. Davis, secretary. One vacancy only on the council occurs each year, and the term of office is five years. This time Dr. L. McL. Tiffany's term expired, and he was reëlected. His home is in Baltimore. The other members of the council are Dr. L. S. McMurtry, of Louisville; Dr. George J. Englemann, of Boston, and Dr. G. M. Johnson, of New Orleans. Dr. Ernest S. Lewis, of New Orleans, was made chairman of the committee on arrangements for the next annual meeting.

---

THE Buffalo Academy of Medicine held meetings during the month of December, 1898, as follows:

*Section on Surgery.*—Tuesday evening, December 6th, program: The curability of cancer, by N. Jacobson, of Syracuse; discussed by Drs. John Parmenter, H. R. Gaylord and others. Colles's fracture, by E. M. Dooley; discussion by Drs. W. C. Phelps, M. Hartwig, E. A. Smith and others.

*Section on Medicine.*—Tuesday evening, December 13th, program: treatment of cases of pulmonary tuberculosis that cannot leave home, by DeLancey Rochester. Abnormalities of the blood, the etiology of asthma, by George N. Jack, Depew, N. Y.

*Section on Pathology.* —Tuesday evening, December 20th, program: The test of renal insufficiency by methylene blue and iodine, by Richard C. Cabot, Boston, Mass. Exhibition of lantern slides of various skin affections, by Grover W. Wende.

*Section on Obstetrics.*—Tuesday evening, December 27th, program: Treatment of placenta previa, by S. S. Greene; discussion, Dr. Van Peyma; specimen of placenta previa, Dr. Brennen. Extra uterine gestation, specimen, Dr. Roemmelt.

